# Observation of a unique case of metastatic basal cell carcinoma found by radiographic evaluation in a patient with oculocutaneous albinism

**DOI:** 10.12688/f1000research.3-10.v1

**Published:** 2014-01-15

**Authors:** Mickaila Johnston, Whitney Winham, Nicole Massoll, Jerad M. Gardner

**Affiliations:** 1 Division of Nuclear Medicine, University of Arkansas for Medical Sciences, Little Rock, AR, 72205, USA; 2 Current address: Naval Medical Center San Diego, CA, USA; 3 Department of Pathology, University of Arkansas for Medical Sciences, Little Rock, AR, 72205, USA

## Abstract

**Background:** Basal cell carcinoma is one of the more common cancers worldwide; 2.8 million are diagnosed annually in the USA.  However, the rate at which it metastasizes is considered very low, between 0.0028 and 0.5%.  For those rare cases in which metastases occur, approximately one third metastasize to the lung.

**Case:** Presented is a 62-year-old Caucasian male with oculocutaneous albinism and a history of basal cell carcinomas occurring in multiple anatomic sites, most recently at the bilateral forearm and back.  Surveillance PET/CT imaging led to the discovery of no less than 30 lung nodules which were consistent with basal cell carcinoma on biopsy.  Histological features were remarkably similar in both the primary tumor and in the metastases.

**Conclusion:**  An unusual case of a non-head and neck primary basal cell carcinoma metastatic to the lung was discovered on surveillance PET/CT imaging, in a patient with oculocutaneous albinism.

## Background

Basal cell carcinoma (BCC) is the most common human malignancy worldwide, yet it is typically indolent and rarely possesses metastatic potential ^1^. Reported rates of metastases range from 0.0028 to 0.5% ^1^. Despite the high incidence of BCC, there have been only 257 cases of metastatic BCC (MBCC) reported in the English medical literature between 1894 and 1991, 82 of which demonstrated metastases to the lung ^2– 5^.

In this article, we review the clinical, radiological, and histopathological presentation of a patient with a history of multiple non-head and neck BCC with subsequent numerous metastases to the bilateral lungs. We also briefly review the literature, and discuss the epidemiology, risk factors, TNM staging, therapeutic modalities, and prognosis for patients with MBCC.

## Case

A 62-year-old Caucasian male with oculocutaneous albinism (Fitzpatrick type I skin) had been followed extensively by both the dermatology and the general surgery services at the University of Arkansas for Medical Sciences. His past medical history was significant for multiple BCCs, the most recent of which (2012) involved the back and flank, requiring adjuvant radiation therapy and split thickness skin grafting. No other significant medical history was noted aside from shortness of breath.

Additionally, four months prior to these excisions, the patient underwent excisions of morpheaform (infiltrative) BCCs of the right arm and back, as well as nodular BCCs of the left cheek and temple. In 2009, he had an initial large wide excision for BCC on his back and flank which demonstrated positive deep margins. The most recent re-excision in 2012 demonstrated all negative margins. Moreover, in 2012 he had a singular squamous cell carcinoma of the right upper extremity that was less than 1.0 mm to the nearest margin, and measured 4.0 mm in maximum depth of invasion.

## Roentgenographic findings

A routine chest X-ray in 2009 was effectively within normal limits, displaying no mass or tumor. A diagnostic CT scan was ordered in 2012 for surveillance due to the extensive nature of his BCCs. It demonstrated numerous solid and sub-solid nodules measuring up to 2.0 cm, in multiple stages of cavitary change, in both lungs. It was considered possible that the nodules were metastases from the squamous cell carcinoma of the right arm but further evaluation was recommended to confirm this.

**Figure 1.**
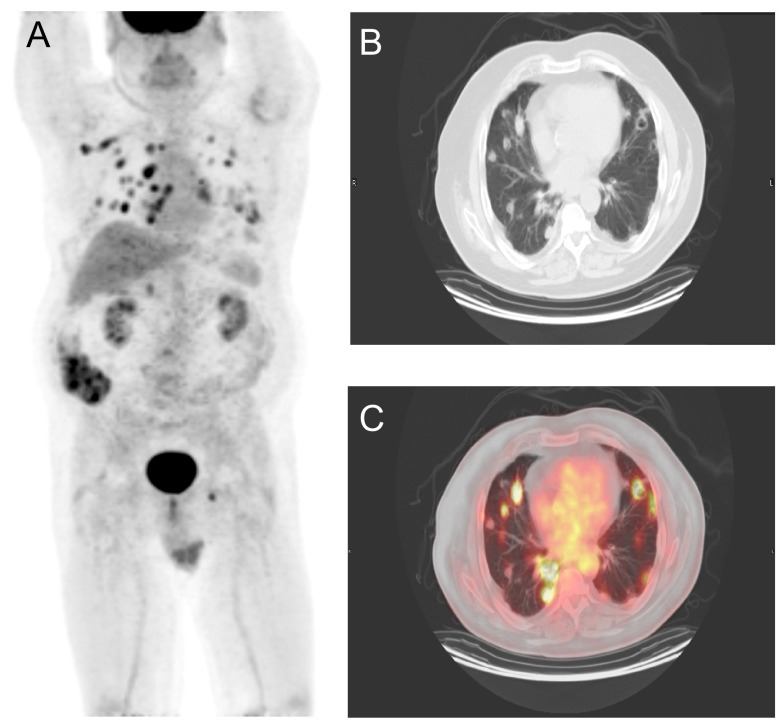
**a**) Maximum Intensity Projection, **b**) axial CT, and **c**) fused PET/CT scans demonstrates multiple hypermetabolic bilateral lung cavitary nodules (foci) in multiple stages of development. This is a non-specific finding, as inflammation and malignancy may present in a similar manner.

Follow-up F-18 fluoro-deoxy-glucose PET/CT scan (15.14 mCi, 69-minutes of uptake time, and a fasting blood glucose of 104 mg/dL) was performed from the base of the orbits through the mid-thigh with 3-axis reconstructions, and attenuation correction with a non-diagnostic CT scan. It demonstrated no less than thirty non-specific foci with significant hypermetabolic activity (>3-times background), most of which were associated with nodules in multiple stages of cavitary change ( Figure 1). In light of the patient's history of multiple malignancies, in addition to an inflammatory/infectious etiology, the possibility of metastasis, although less likely, was also considered. Clinical and pathologic correlations were recommended, as well as a repeat PET/CT of the vertex through the feet, for definitive evaluation of the dermis.

## Histopathologic findings

**Figure 2.**
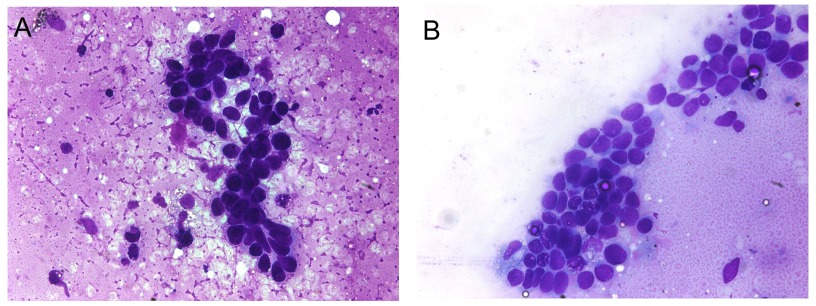
Fine needle aspiration (Diff-Quick preparation, original magnification ×400) from a lung nodule demonstrates **a**) Cells with a high nuclear to cytoplasmic ratio and mildly enlarged nuclei compared to surrounding inflammatory cells in the background, **b**) Enlarged, fairly uniform cells forming sheets.

CT-guided fine needle aspiration and subsequent core biopsy from one of the lung nodules from the right upper lobe were interpreted as positive for malignant cells, basal cell carcinoma. The right upper lobe core biopsy showed small cohesive nests and cords of basaloid cells with scant cytoplasm. Artifactual clefts containing mucin were present around the periphery of many of the nests. The cells demonstrated hyperchromatic chromatin without nucleoli and displayed some nuclear overlap and molding. The tumor cells were negative for synaptophysin, chromogranin, and cytokeratin 20 by immunohistochemistry, findings which argue against the possibility of primary or metastatic neuroendocrine carcinoma ( Figure 2).

**Figure 3.**
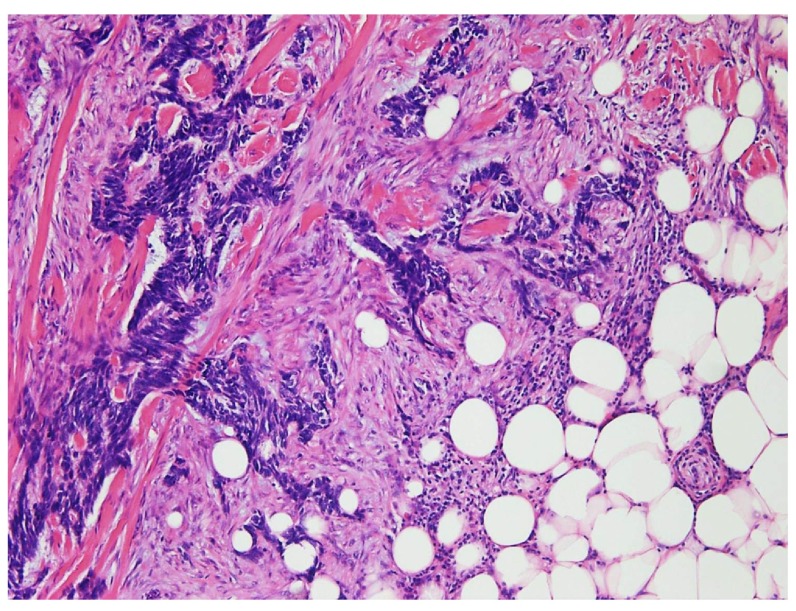
Right flank excision demonstrates infiltrative cords and strands of basaloid cells in a desmoplastic stroma characteristic of infiltrative type of BCC (hematoxylin-eosin stain, original magnification ×100).

A right flank wide local re-excision from 2012, several months prior to the discovery of lung metastases, demonstrated infiltrative cords and strands of basaloid cells characteristic of the infiltrative (morpheaform) type of basal cell carcinoma. Multiple tumor nodules were present in this specimen and the tumor invaded into the subcutis. Margins were negative. This right flank basal cell carcinoma specimen was re-examined and immunohistochemical stains were performed following discovery of the lung metastases; the tumor cells were negative for synaptophysin, chromogranin, and cytokeratin 20 by immunohistochemistry, findings which argue against the possibility of a cutaneous neuroendocrine carcinoma (Merkel cell carcinoma), ( Figure 3).

Biopsies and excisions from the scalp, left temple, right arm, and back performed several years previously were also reviewed and all were found to show characteristic histologic features of basal cell carcinoma.

The histologic features in the lung biopsy strongly resembled the histologic features of the multiple infiltrative basal cell carcinomas that this patient has had previously. The similarity between the tumors, the history of multiple aggressive and deeply invasive BCCs, and the exclusion by immunohistochemistry of the histologic mimics are all features that suggest that the pulmonary nodules represent a very rare example of metastatic basal cell carcinoma.

## Treatment and follow-up data

The patient was started on vismodegib, the cyclopamine-competitive antagonist of the smoothened receptor, at a dosage of 150 mg by mouth each day. After approximately two months of therapy, he began to have an improvement in his ability to breathe, and he was able to self-taper off of his home supplemental oxygen requirement. A follow up CT scan at this time showed disease improvement, and the vismodegib was discontinued. Most recently, a follow up CT, seven months after initial diagnosis of pulmonary metastases, showed interval worsening of the metastatic burden in the lungs. He is scheduled to restart vismodegib and is now being followed jointly by the palliative care and medical oncology services.

## Discussion

In 1894, Beadles described a singular case of “rodent ulcer” deposition within the lymphatic gland of a deceased 46-year-old male, who had a previous history of “rodent ulcer” of the cheek. At autopsy, caseating foci were found within his lungs ^3^. Although the historical medical terminology precludes absolute certainty, this is thought to represent the first reported case of metastatic basal cell carcinoma (MBCC).

Lattes and Kessler used three criteria to define MBCC in their 1951 report of two cases. First, they required that neither the primary nor the metastases could be squamous in type. Second, tumors could not be considered new primaries or a result of direct extension. Third, the tumors could not be mucoid or salivary in origin ^5^.

In total, there have been only 257 cases of MBCC reported in the literature between 1894 and 1991 (based on a PubMed search using the term “metastatic+basal+cell+carcinoma” and adding the number of cases presented in each article)”.

Metastases are thought to arise from both lymphatic and hematogenous pathways ^1^. The most frequent reported sites of metastases were regional lymph nodes (60–65%), lung (32.3–40%), bone (19–25.8%), and skin (10–19%) ^2, 4^.

In light of the rarity of MBCC, it is unclear which histological or clinical features might be correlated with the risk of metastasis. With regards to histological features, there is a correlation with increased risk of local recurrence in specific subtypes like morpheaform or infiltrating types, but no correlation has been found with an increased risk of metastases among these more locally aggressive subtypes ^6^. Of the 257 cases reported between 1894 and 1991, only five cases of MBCC were in black patients -the remainder were all in patients with a light complexion ^4^. Disorders such as Basal Cell Nevus Syndrome or Xeroderma Pigmentosum have been shown to predispose patients to BCC. However, none have been shown to have a higher rate of metastases ^7, 8^. Patients with specific genodermatoses such as oculocutaneous albinism, in which there is a disorder of the melanin biosynthesis, have been shown to have a much less specific association with BCC. The significance of this association is unknown at this time ^8^. However, a history of radiation therapy and history of local tumor recurrence have both been implicated in higher rates of MBCC ^8^. In 170 of the reported cases of MBCC, the most frequent primary sites were head (67.6%) and trunk (16.5%), anatomic locations which are not significantly different than the most frequent sites of typical non-metastatic BCC ^1^. In a review of 41 publications by Snow *et al.*, BCCs larger than 4.0 cm had a 1.9% chance of metastases ^5^. However, primary BCCs as small as 1.1 cm have been reported to metastasize ^9^.

Survival time after the development of distant metastases in MBCC has been reported at 8–10 months ^2, 4^. A median interval between discovery of the primary focus of BCC and the discovery of metastasis has been reported as 9 years ^2^, much longer than the interval seen in many other types of carcinoma.

For surveillance of patients with only local BCC, close follow up with a thorough skin examination, loco-regional lymph node evaluation, periodic Roentgenographic evaluation, liver function tests and alkaline phosphatase tests have been suggested to evaluate for occurrence of distant metastases ^10, 1^. However, given the extreme rarity of metastasis from BCC, these suggestions may not be feasible or reasonable for most patients. Further studies to elucidate risk factors for metastasis in BCC would be useful in determining which patients should receive a higher level of follow up screening.

As with many rare diseases, there is no established standard of care for management of metastatic foci in MBCC. A variety of therapies have been reported in the literature including local excision, radiation therapy, and chemotherapy. Given the aforementioned short survival times, large BCCs (>5 cm) and multiple metastatic sites pose a difficult dilemma for treatment, surgery may result in functional and cosmetic impairment, and radiation therapy is poor at providing local control ^12^.

For metastatic disease, debulking prior to local surgery, and for a failure of local treatment, cis-platinum containing regimens had been a commonly accepted therapeutic approach ^12^. However, preliminary results from NCT00833417 [a study evaluating the efficacy and safety of vismodegib (GDC-0449, Hedgehog pathway inhibitor) in patients with advanced basal cell carcinoma] prompted the USFDA to approve vismodegib as a treatment for “adults with BCC, that has spread to other parts of the body, or that has come back after surgery, or that their healthcare provider decides cannot be treated with surgery or radiation”, on January 30, 2012.

In the present patient, with history of oculocutaneous albinism, there were in excess of 30 metastatic foci within the lungs, whose histologic features strongly resembled those of the patient’s multiple other infiltrative BCCs. The primary tumor as well as the metastases were not squamous in type. Also, the squamous cell carcinoma of the arm was well differentiated and did not demonstrate basaloid features. Because primary basal cell carcinomas do not arise in the lungs and there was no evidence of direct extension from the back and flank into the lung tissue, the lung lesions are considered to be true metastases, as evidenced by the multiple lung nodules. Lastly, none of the primary or metastatic lesions of BCC demonstrated any mucoid or salivary features. Given the immunohistochemical exclusion of histologic mimics and history of multiple deeply invasive and aggressive BCCs it is considered most likely that the lung based cavitary foci were MBCC. Neuroendocrine primary tumors of the lung were systematically ruled out by immunohistochemical stains and the previous skin resections were ruled out for Merkel cell involvement.

Because of the tumor burden involving the lungs, surgery was not considered an option. He was treated with a two month course of vismodegib, to which he initially responded well. Surveillance CT scans and clinical examinations are currently being used to determine the need for resumption of vismodegib. Secondary to a lack of prospective studies or large sample sizes of previous studies, it remains unclear if the oculocutaneous albinism of the present patient was simply an association or a predisposing factor.

In conclusion, an unusual case of MBCC is presented that is presumed to arise from a non-facial primary BCC, with metastasis to the lung, detected by radiographic interrogation. It is important for providers to be aware that BCC may rarely metastasize, and that the risk for metastasis may be higher in patients with a primary BCC that is >4 cm or that has been previously irradiated. Imaging specialists should be sure to keep MBCC in the differential diagnosis when faced with a lung, lymph node, or cutaneous focus that is hypermetabolic and there is a history of BCC.

## Consent

No consent was obtained from the patient. The case report is fully anonymized and HIPAA-compliant, contains no patient identifiers in either the text or the figures, and was performed as an IRB exempt study.

## References

[1] Soleymani AD Scheinfeld N Vasil K: Metastatic basal cell carcinoma presention with unilateral upper extremity edema and lymphatic spread. *J Am Acad Dermatol.* 2008;59(2 Suppl 1):S1–S3 10.1016/j.jaad.2007.08.041 18625368

[2] von Domarus H Stevens PJ: Metastatic basal cell carcinoma. Report of five cases and review of 170 cases in the literature. *J Am Acad Dermatol.* 1984;10(6):1043–1060 10.1016/S0190-9622(84)80334-5 6736323

[3] Beadles CF: Rodent ulcer. *Trans Pathol Soc Lond.* 1894;45:176–181

[4] Lo JS Snow SN Reizner GT: Metastatic basal cell carcinoma: report of twelve cases with a review of the literature. *J Am Acad Dermatol.* 1991;24(5 Pt 1):715–719 10.1016/0190-9622(91)70108-E 1869642

[5] Snow SN Sahl W Lo JS: Metastatic basal cell carcinoma: report of five cases. *Cancer.* 1994;73(2):328–335 10.1002/1097-0142(19940115)73:2<328::AID-CNCR2820730216>3.0.CO;2-U 8293396

[6] Kogan L Ariely D Pizov G: Metastatic spinal basal cell carcinoma: a case report and literature review. *Ann Plast Surg.* 2000;44(1):86–88 10.1097/00000637-200044010-00016 10651373

[7] Nikolaou V Stratigos AJ Tsao H: Hereditary nonmelanoma skin cancer. *Semin Cutan Med Surg.* 2012;31(4):204–210 10.1016/j.sder.2012.08.005 23174490 PMC3759014

[8] Castori M Morrone A Kanitakis J: Genetic skin disease predisposing to basal cell carcinoma. *Eur J Dermatol.* 2012;22(3):299–309 10.1684/ejd.2011.1633 22391625

[9] Lattes R Kessler RW: Metastasizing basal-cell epithelioma of the skin; report of two cases. *Cancer.* 1951;4(4):866–878 10.1002/1097-0142(195107)4:4<866::AID-CNCR2820040424>3.0.CO;2-F 14859207

[10] Berlin JM Warner MR Bailin PL: Metastatic basal cell carcinoma presenting as unilateral axillary lymphadenopathy: report of a case and review of the literature. *Dermatol Surg.* 2002;28(11):1082–1084 10.1046/j.1524-4725.2002.02090.x 12460309

[11] Malone JP Fedok FG Belchis DA: Basal cell carcinoma metastatic to the parotid: report of a new case and review of the literature. *Ear Nose Throat J.* 2000;79(7):511–519 10935303

[12] Pfeiffer P Hansen O Rose C: Systemic cytotoxic therapy of basal cell carcinoma: A review of the literature. *Eur J Cancer.* 1990;26(1):73–7 10.1016/0277-5379(90)90262-R 2138485

