# Supplementary material for: Observation of a unique case of metastatic basal cell carcinoma found by radiographic evaluation in a patient with oculocutaneous albinism
Source: F1000Res. 2014 Jan 15;3:10. [Version 1] doi: 10.12688/f1000research.3-10.v1 (PMC3901453; doi:10.12688/f1000research.3-10.v1)
Supplement: Peer review history

# Peer review: Referee response for version 1

**Published:** 2014-01-23

**Authors:** Angela Bohlke

**Affiliations:** 1 Dermapathology and Aesthetics, Silver Falls Dermatology, Salem, OR, USA

I found this to contain a thorough review of literature with case-specific data and analysis that will contribute to future cases. The case was worked up well and the review of it complete. The outcome of it may also help guide future management and prognosis.

I have read this submission. I believe that I have an appropriate level of expertise to confirm that it is of an acceptable scientific standard.

# Peer review: Referee response for version 1

**Published:** 2014-01-22

**Authors:** Jordan Reynolds

**Affiliations:** 1 Department of Anatomic Pathology, Cleveland Clinic, Cleveland, OH, USA

The authors present a unique case of metastatic BCC to the lungs in a patient with oculocutaneous albinism. Briefly; the patient had a history of multiple subtypes of BCC and had a SCC of the upper extremity. He presented with lung nodules on imaging and underwent biopsy FNA to elucidate whether the lesions arose from BCC or SCC. Morphologic and immunophenotypic evidence supported the former. The authors then go on to provide a detailed summary of the history of metastatic BCC and risk factors that may increase the chance for metastasis, including cutaneous syndromes. The authors also discuss current treatment regimens for these patients. This paper has value for pathologists, dermatologists and oncologists as it presents a unique entity (metastatic BCC) in a patient with an interesting medical co-morbidity.

I do think the paper needs major revisions however. Everything that is written is written well and needs no major changes but the pictures have major issues. The cytology pictures (figure 2) do not look like tumor cells. They may be tumor cells, but they really look like reactive mesothelial cells. If this was a transthoracic biopsy, they very well could be reactive mesothelial cells. Also, there are no pictures of the core biopsy. The authors need better pictures of the cyto and need to add the core biopsy photos, OR get rid of the cyto pictures completely, and add some high quality images of the core biopsy and pertinent immunostains.

I have read this submission. I believe that I have an appropriate level of expertise to confirm that it is of an acceptable scientific standard, however I have significant reservations, as outlined above.

# Peer review: Referee response for version 1

**Published:** 2014-01-17

**Authors:** Amanda Mullins

**Affiliations:** 1 Yosemite Pathology Medical Group, Emanuel Medical Centre, Turlock, CA, USA

This is an interesting report of a rare phenomenon.

I have read this submission. I believe that I have an appropriate level of expertise to confirm that it is of an acceptable scientific standard.
